# High circulating hepatocyte growth factor levels associate with epithelial to mesenchymal transition and poor outcome in small cell lung cancer patients

**DOI:** 10.18632/oncotarget.2124

**Published:** 2014-06-20

**Authors:** Israel Cañadas, Álvaro Taus, Iria González, Xavier Villanueva, Javier Gimeno, Lara Pijuan, Manuel Dómine, Albert Sánchez-Font, Ivan Vollmer, Silvia Menéndez, Oriol Arpí, Sergi Mojal, Federico Rojo, Ana Rovira, Joan Albanell, Edurne Arriola

**Affiliations:** ^1^ Cancer Research Program, IMIM (Hospital del Mar Medical Research Institute), Doctor Aiguader, Barcelona; ^2^ Servei d'Oncologia Médica, Hospital del Mar, Passeig Marítim, Barcelona; ^3^ Servei de Patologia, Hospital del Mar, Passeig Marítim, Barcelona; ^4^ Oncology Department, IIS-Fundación Jiménez Díaz, Avenida Reyes Católicos, Madrid; ^5^ Servei de Pneumologia, Hospital del Mar, Passeig Marítim, Barcelona; ^6^ Servei de Radiologia, Hospital del Mar, Passeig Marítim, Barcelona; ^7^ Consulting Service on Methodology for Biomedical Research, IMIM, Doctor Aiguader, Barcelona; ^8^ Pathology Department, IIS-Fundación Jiménez Díaz, Avenida Reyes Católicos, Madrid; ^9^ Pompeu Fabra University, Doctor Aiguader, Barcelona

**Keywords:** Small Cell Lung Cancer, Hepatocyte Growth Factor, Met, Epithelial to Mesenchymal Transition, Chemoresistance

## Abstract

We have previously shown that Met activation through the hepatocyte growth factor (HGF) increases tumorogenesis, induces epithelial-to-mesenchymal transition (EMT) and chemoresistance in SCLC. We sought to evaluate circulating HGF levels in SCLC patients and assess correlation with outcome and EMT features in the tumor. Serum samples from patients with SCLC were prospectively obtained at diagnosis, response evaluation and progression. HGF serum (sHGF) was quantified by ELISA. EMT markers and p-Met/Met were assayed by immunohistochemistry in tumor samples. Clinical data were prospectively recorder. One-hundred twelve patients were included. High baseline levels of sHGF were associated with shorter overall survival (p=0.007) and remained independently associated with survival in the multivariate analysis (p=0.016). For stage IV patients, an increase of sHGF levels at response evaluation (p=0.042) and at progression (p=0.003) were associated with poor outcome. sHGF levels were associated (p<0.05) with a mesenchymal phenotype in the tumor. In conclusion, high sHGF at diagnosis and increases during the course of the disease predict for poor outcome in SCLC patients and associate with EMT in the tumor. These data provide novel evidence on a role of sHGF in the adverse clinical behavior of SCLC and support testing Met inhibitors in patients with high sHGF.

## INTRODUCTION

Small cell lung carcinoma (SCLC) is a highly lethal disease and accounts for approximately 15% of patients with lung cancers[1]. Many genetic alterations have been identified with potential therapeutic interest [2-4]. However, no targeted treatment has been successful to date in improving the outcome of patients. Outcome in advance stage remains poor with a median overall survival that does not exceed one year with available treatments [5]. The research of novel targets for selected patient populations in this disease is therefore urgently needed.

Met is a transmembrane receptor tyrosine kinase that is overexpressed in many solid tumors and has been associated with poor outcome. Hepatocyte growth factor (HGF) is the high affinity natural ligand of Met and upon binding to the receptor, it triggers dimerization of the receptor and downstream signaling. Aberrant Met activation through HGF (autocrine or paracrine effects) or genetic mechanisms (mutation, amplification) is associated with increased motility, migration, invasion and angiogenesis in several tumor models[6-8]. A number of Met inhibitors are in development at the moment with promising results in solid tumors[9].

We have previously reported that Met activation as assayed by phosphorylated Met (p-Met) expression is associated with decreased survival in SCLC[10]. We have also shown in preclinical SCLC models that HGF induces epithelial to mesenchymal transition (EMT) that results in increased tumorogenesis, invasiveness and chemoresistance. The potential clinical relevance of this finding was further suggested by the ability of Met inhibition, achieved by the Alk/Met inhibitor crizotinib, to re-sensitize mesenchymal SCLC tumor xenografts to chemotherapy. In human SCLC samples we have also observed an association between Met activation and mesenchymal markers (vimentin, Snail1, SPARC) and poor outcome. Furthermore, mesenchymal features were upregulated in relapsed, chemorefractory disease [11]. Studies have also shown an association between EMT features in the tumor and outcome for NSCLC[12]. These data provide rational to consider clinical trials combining chemotherapy with Met inhibitors in SCLC patients with a mesenchymal/Met activated phenotype.

The hypothesis of the present work was that circulating HGF would be a clinically useful surrogate marker of EMT and Met phenotype in SCLC and therefore correlate with patient outcome. Serum HGF (sHGF) has been associated with prognosis in several tumors [13-16], and response/resistance to therapies [17-19]. Ultimately, if this were the case in SCLC, then it could be considered as a potential biomarker for defining a population to be tested with Met inhibitors.

Peripheral blood and its components (serum, plasma, and circulating cells) provide a non-invasive medium to evaluate biomarkers in a more convenient way for patients compared to a lung biopsy. It also allows for serial determinations of the biomarker and correlation with treatment effects.

## RESULTS

### SCLC patients have higher HGF serum levels when compared to healthy subjects

We included 112 SCLC patients in this study. Patients’ characteristics are shown in Table 1. As observed, the majority were male, current smokers with good performance status (PS). The metastatic locations were as expected with a majority of patients having liver and bone disease. First line treatment was standard chemotherapy with a higher percentage of patients receiving carboplatin (70%) in combination with etoposide. Patients that were considered unfit for treatment underwent best supportive care. This particular group of patients (N: 9) were characterized by poor PS (2-4) and only had the baseline sHGF sample.

**Table 1:** Patients’ characteristics

N: 112			N(%)
Median age (range)			66 (29-90)
Gender	Male		89 (79)
	Female		23 (21)
Smoking history	Current		83 (74)
	Former		29 (26)
PS	0-1		77 (69)
	2-4		35 (31)
Stage	I-III		30 (27)
	IV		82 (73)
Metastatic location*	Lung		9 (11)
	Pleura		18 (22)
	Liver		31 (38)
	Bone		25 (30)
	Adrenal		23 (28)
	CNS		20 (24)
Chemotherapy	Cisplatin		33 (30)
	Carboplatin		69 (62)
	None		9 (8)

* percentages considering stage IV patients (N:82)

As differences between serum and plasma levels of HGF have been reported we first analyzed 26 cases with both types of samples. Supplementary figure 1 shows the correlation between both types of samples with higher levels found in serum as expected [20, 21].

**Figure 1:**
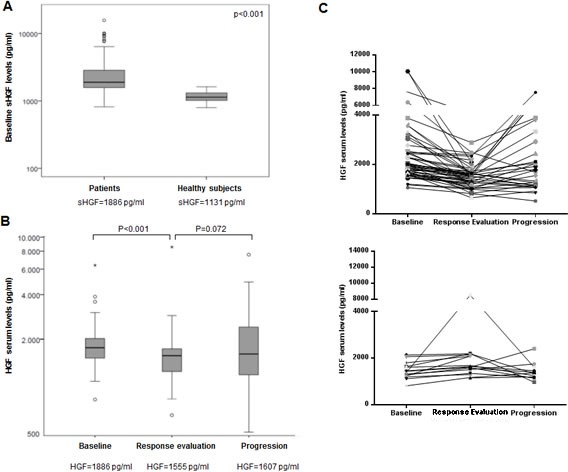
Serum HGF levels change longitudinally in patients with SCLC undergoing chemotherapy (A) Baseline serum hepatocyte growth factor (sHGF) levels are elevated in SCLC patients when compared to healthy subjects. sHGF levels at diagnosis were measured in SCLC patients and healthy subjects using a ELISA kit. Box plot is displayed indicating sHGF levels in each group. sHGF medians are indicated below. Wilcoxon test was used for comparisons. (B) sHGF levels change in SCLC patients after treatment and at progression. Whole-blood samples were obtained from each SCLC patient during different disease stages (baseline, chemotherapy response assessment and progression). sHGF levels were determined by ELISA kit. Box plot is displayed indicating sHGF levels at different time points. sHGF medians are indicated below. Wilcoxon test was used for comparisons. (C) Graphs represent sHGF levels for each SCLC patient during different time points. Top: patients who show a decrease at response evaluation; bottom: patients who show an increase at response evaluation.

We then collected serum from 30 healthy volunteers matched to the study population by smoking status, gender and age. sHGF levels were variable in healthy subjects ranging from 792 to 1618 pg/ml, with a median sHGF of 1131 pg/ml. sHGF levels for SCLC patients (N:104) at diagnosis were significantly higher than in healthy volunteers with a median of 1886 pg/ml (p<0.001). The range of levels was greater in patients showing values from 816 to 15629 pg/ml(Figure 1A).

Next, we evaluated if sHGF was associated with any of the clinical variables. We found a significant association between higher sHGF and worse PS (2-4) (p=0.002), and presence of liver metastases (p=0.006). This was consistent when we evaluated sHGF as a continuous variable or dichotomous divided by the median (1886pg/ml).

### sHGF levels change longitudinally in patients with SCLC undergoing chemotherapy

Figure 1B illustrates sHGF levels at diagnosis (N:104) when compared to sHGF at response evaluation (N:72) and at first progression (N:51). At response evaluation, after 2-3 cycles of chemotherapy, in 78% of the cases sHGF showed a decrease. sHGF levels at this point were significantly lower than at baseline with a median of 1555pg/ml (p<0.001). Moreover, at first clinically detected progression, 58% patients showed increase of sHGF from the response evaluation time point (p=0.072) and 48% if compared to baseline (p=0.76). The median value at progression was 1607pg/ml. Changes for each individual patient are plotted in Figure 1C.

### sHGF levels at diagnosis and changes during treatment have an impact in prognosis

We assessed the impact of all clinical variables on survival in univariate analysis. Median follow up for the series was 7.2 months (0.1-166.4). Table 2 illustrates the association between clinical variables and OS. Median overall survival for the whole series was 9.5 months. Increasing age (p=0.002), poor PS, and former smoking history were associated with decreased survival (Table 2). The fact of not receiving treatment was also significantly associated with poorer survival. Within stage IV patients, no differences were observed between patients receiving cisplatin or carboplatin. Regarding location of metastases, those with pleural (p=0.059) or liver metastases (p=0.002) showed decreased survival.

**Table 2:** Univariate analysis between clinical variables and overall survival (Cox regression model)

		OS (months)	HR (95%CI)	p-value
Gender	Male	9.23 (5.27-13.20)	1.24 (0.68-2.25)	0.488
	Female	11.63 (8.63-14.63)		
Tobacco history	Current	14.82 (9.55-20.08)	2.31 (1.36-3.93)	0.002
	Former	6.77 (1.78-11.76)		
PS	0-1	14.82 (11.77-17.87)	3.64 (2.18-6.09)	<0.001
	2-4	3.81 (0.96-6.66)		
Treatment	Yes	11.70 (8.48-14.91)	0.061 (0.024-0.15)	<0.001
	No	0.76 (0.84-1.43)		
Stage	I-III	33.25 (19.26-47.24)	4.18 (2.05-8.50)	<0.001
	IV	8.80 (7.49-10.11)		
Response to first line	Yes	12.32 (8.95-15.69)	1.77 (0.75-4.22)	0.194
	No	6.21 (1.31-11.10)		
sHGF (pg/ml)	<1886	12.58 (6.03-19.14)	2.02 (1.21-3.37)	0.007
	>=1886	7.75 (5.11-10.39)		

**Figure 2:**
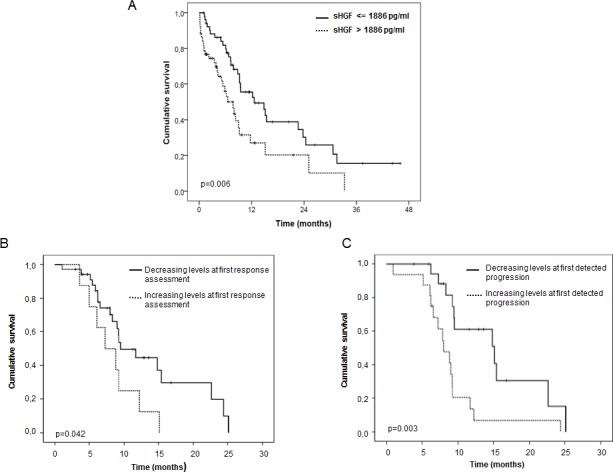
sHGF levels at diagnosis and changes during treatment are predictive of outcome in SCLC patients (A) Kaplan-Meier curve for overall survival according to baseline sHGF levels in SCLC patients. The median sHGF level at diagnosis (1886 pg/ml) was used as the cut-off. P-values were calculated using the log-rank test. Significance is displayed. (B) Kaplan-Meier curve for overall survival according to changes (increase vs decrease) in sHGF levels at response evaluation (first CT scan) in stage IV SCLC patients. P-values were calculated using the log-rank test. Significance is displayed. (C) Kaplan-Meier curve for overall survival according to changes (increase vs decrease) in sHGF levels at first clinically detected progression in stage IV SCLC patients. P-values were calculated using the log-rank test. Significance is displayed.

We next evaluated the impact of sHGF levels at baseline on outcome of these patients. Patients’ characteristics in both sHGF high and sHGF low groups are shown in Table 3. Higher levels of sHGF were associated with worse survival when analyzed as both continuous or as discrete variable (median as the cut-off). Figure 2A shows the Kaplan-Meier curve for survival depending on sHGF levels (Table 2). Increases in 1000 pg/ml were associated with a HR: 1.28 (1.14-1.42) (p<0.001) of dying. This association was also significant when analyzing only stage IV patients (HR: 1.29 (1.14-1.46), p= 0.001).

**Table 3:** Patients’ characteristics according to levels of sHGF

Patients’ characteristics		sHGF low* N (%)	sHGF high** N (%)	p-value
Age	Mean (SD)	65.3 (10.4)	66.6 (10.8)	0.52
Gender	Male	39 (75)	43 (82.7)	0.34
	Female	13 (25)	9 (17.3)	
Tobacco history	Current	41 (78.8)	37 (71.2)	0.36
	Former	11 (21.2)	15 (28.8)	
PS	0-1	43 (82.7)	28 (53.8)	0.002
	2-4	9 (17.3)	24 (46.2)	
Treatment	Yes	49 (94.2)	45 (88.2)	0.32
	No	3 (5.8)	6 (11.8)	
Stage	I-III	17 (32.7)	11 (21.2)	0.18
	IV	35 (67.3)	41 (78.8)	

sHGF: serum Hepatocyte Growth Factor; SD: standard deviation; N: number

* sHGF levels <=1886.10 pg/ml;

** sHGF levels >1886.10 pg/ml

In addition, we also performed the analysis excluding those patients that did not receive treatment, that included all patients with PS:4 (and thus would not be eligible for any therapy, including the suggested Met inhibitors). Again, those patients with sHGF above the median showed a statistically significant worse survival than those with lower sHGF (7.9 months vs 14.8 months, respectively; p=0.023).

We then performed a multivariate analysis including all significant variables in univariate analysis. Table 4 shows the results of the Cox regression model with the variables that remained independently associated with OS. As observed, higher sHGF levels were associated with survival in this model as well as poor PS, former smoking history, advanced stage, the presence of pleural metastases and the lack of treatment.

**Table 4:** Multivariate Cox regression model for OS

	HR	CI (95%)	p-value
PS (2-4 vs 0-1)	3.56	(1.89-6.67)	<0.001
Treatment (Yes vs No)	0.16	(0.06-0.44)	<0.001
Stage (IV vs I-III)	2.98	(1.31-6.79)	0.009
Pleural Mets (Present)	2.05	(1.01-4.14)	0.047
Tobacco (former vs current)	1.96	(1.05-3.67)	0.035
sHGF (>=1886 pg/ml)	1.94	(1.13-3.31)	0.016

Then we evaluated if changes of sHGF during treatment were associated with OS. We selected stage IV patients because treatment (chemotherapy alone) and outcomes were more homogeneous in this subgroup. Patients who had a decrease of sHGF from baseline to response evaluation presented a longer OS (9.5months) compared to those that experimented an increase in sHGF at this time point (7.3months) (p: 0.042) (Figure 2B). Moreover, those patients whose sHGF levels increased from baseline to progression presented shorter survival (8.9 months) vs those whose sHGF was lower at progression (15.2 months) (p: 0.003) (Figure 2C).

In order to investigate the potential associations between sHGF levels and response with the impact in survival, we evaluated the association of sHGF levels and its changes with tumor response. We had available information on response to treatment in 89 patients. From these, 6 (7%) patients showed complete response, 75 (84%) partial response, 5 (6%) stable disease and 3 (3%) progression. These categories were not associated with overall survival in a statistically significant manner, although numerically, patients who responded doubled OS compared to those who did not (p=0.194) (Table 2). No significant correlation was found between response and baseline sHGF levels (median sHGF levels in responders: 1793pg/ml vs non-responders: 1917pg/ml). Moreover, sHGF variations during treatment (i.e. increase or decrease at response evaluation and progression) were not associated with response either. These observations may be related to the very high percentage of responders in these series.

### Serum HGF levels correlate with EMT phenotype in the tumor

**Figure 3:**
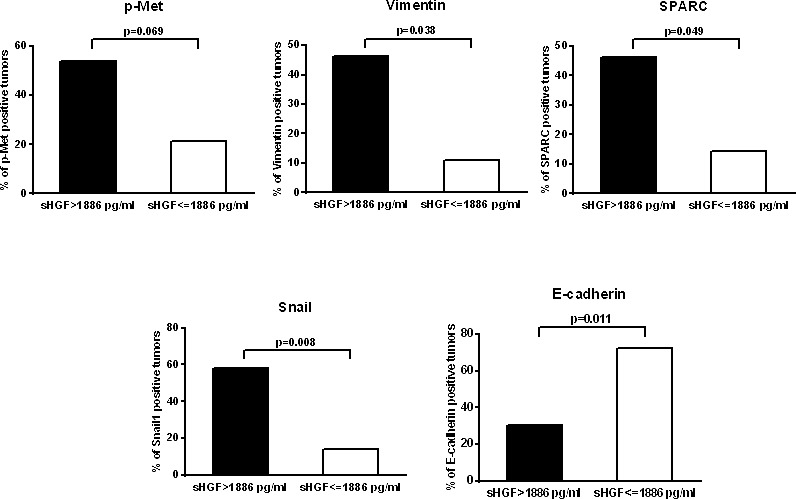
sHGF levels correlate with EMT markers in the tumor Graphs represent the percentage of SCLC tumors positive for EMT markers and p-Met according to HGF serum levels at diagnosis. P-values were calculated using the log-rank test. Significance is displayed.

For a subset of the study population we had enough available tumor samples for tumor biomarker analysis. We had previously shown the induction of EMT through Met activation via HGF in SCLC models and the prognostic impact of these markers in human SCLC [23]. Thus we tested the association between sHGF levels and these tumor markers. We were able to assess these markers in 43 cases for vimentin and Snail1, 44 for SPARC, and p-Met, and 45 for MET and E-cadherin. Some (N:25) of these cases were included in our previous publication[11]. The percentage of positive cases for each marker is shown in Supplementary Table 1. These results are consistent with our previous work showing around 20-30% of SCLC tumors staining for p-Met, vimentin, Snail1 and SPARC and around 50% of cases considered overexpressed (median as cut-off) for Met and E-cadherin. Interestingly, we observed a significant association between increased baseline levels of sHGF (above the median) and Snail1 (p=0.008), vimentin (p=0.038), SPARC (p=0.049) expression and lack of E-cadherin expression (p=0.011). P-Met expression showed a trend towards association with sHGF expression but it did not reach statistical significance (p=0.069) (Figure 3).

## DISCUSSION

SCLC remains one of the neoplasms with less available efficacious treatments, leading to early death of the majority of patients. Many studies are being conducted with targeted therapies, however correlative biomarker studies are still lacking in the majority of the cases. Our study shows an independent role of serum HGF levels in predicting outcome in patients with SCLC. Patients with higher sHGF had shortened survival and an incremental risk for death was found with increasing levels of sHGF at diagnosis. Importantly, sHGF levels were associated with a mesenchymal phenotype in the tumors, a finding that is consistent with prior reports on the ability of the HGF/Met receptor system to induce an epithelial to mesenchymal transition. These results are consistent with a biological relevance of circulating HGF and add to previous evidence supporting the potential role of Met in this lethal disease.

Here we show that sHGF levels in SCLC patients were able to discriminate patients with poor prognosis upfront in line with reports in other tumors[16]. Moreover, changes of serum levels during treatment also predicted for outcome in our series of SCLC patients. The fact that the majority of patients had a decrease of sHGF levels at response evaluation (where the majority of patients do actually respond to treatment) supports the hypothesis of HGF being secreted, at least in part, by tumor cells in an autocrine manner, as described in other tumor models[25]. We have previously demonstrated that SCLC cell lines are able to secrete HGF and this leads to a more mesenchymal phenotype. This would be concordant to what we observe in the current clinical study.

One caveat of the study is that we cannot rule out the predictive role of sHGF in regards to chemotherapy over a prognostic value (lacking a placebo arm as control). We have analyzed specifically the association between tumor response and sHGF levels but we did not find a significant association. Therefore, the predictive role of sHGF regarding chemotherapy response does not seem to be the explanation for the association with patient survival.

A potential implication of our results is in helping to select patients for clinical trials with anti-Met therapies, where the need of predictive biomarker is urgent. Trials with anti-Met therapies have selected patients based on Met immunohistochemistry with an arbitrary cut-off [26]. Although this is a practical approach to conduct a trial that enriches for a population potentially sensitive to anti-Met treatment, it is likely insufficient to optimally select Met sensitive patients. This may help to explain median survival figures around 12 months [26], different from what we find in other target driven tumors where sensitive patients are clearly defined (i.e. EGFR, ALK)[27, 28]. Furthermore, there are examples of Met driven tumors with nice responses to anti-Met therapies. Met amplification seems to be up to date, the best predictor of response to Met inhibitors [29-31]. However, evaluation of copy number changes by FISH is challenging in SCLC due to the typical crushing phenomenon of these tumors and was not feasible in our hands[32]. In a case more relevant to our discussion high sHGF levels appeared to select anti-Met sensitive tumors. In this report, a patient with gastric cancer that exhibited one of the highest baseline circulating HGF from a series of patients treated in a phase I trial with onartuzumab, experienced a complete and durable response [33]. Of note, this was the only patient in the phase I study that had a rapid and sustained decrease in sHGF during anti-Met treatment [20]. This preliminary clinical information, together with extensive preclinical data, raise the hypothesis that selecting patients with ‘active’ Met pathway may improve the efficacy of anti-Met therapies.

It could be argued that the definition of “high” or “low” sHGF based on a unique study is difficult to extrapolate or to incorporate in the clinical practice as a prognostic/predictive factor. Circulating HGF is composed by different isoforms of HGF with differences in their functions [34, 35]. Moreover, differences in sHGF levels according to the detection technique are observed in SCLC studies [22, 36]. Taken together, choosing a cut-off to classify patients as bad prognosis or to select patients for Met inhibitor trials needs further validation with detection techniques that are reproducible and that are predefined in the conception of these trials. However, what seems to be consistent is that increasing levels of HGF are associated with worse survival and may potentially be defining a population of patients with tumors more dependent on Met.

A relevant finding in our study is that, despite the limited number of cases, there was clear association between sHGF levels and mesenchymal biomarkers in the tumor. We have previously shown that p-Met expression is associated with poor survival in SCLC. We subsequently demonstrated that p-Met was co-expressed with EMT markers such as Snail1 and vimentin and these were independently associated with survival. In preclinical models we and others have shown that HGF was able to induce a mesenchymal phenotype (Snail1, SPARC, vimentin expression, E-cadherin absence)[11, 37]. The impact on prognosis of these markers was not evaluated due to the limited sample size.

The clinical data that we present here supports the ability of a sHGF to predict for a mesenchymal status of the tumor, which is associated to Met activation. And this is of upmost potential importance to select these patients for clinical trials with anti-Met therapies and monitor response. Of practical importance, a serum biomarker would be predictive of the status of the tumor and make clinical decisions possible based on a blood test. However, in our study p-Met expression did not statistically correlate with sHGF status, although there was a clear trend (p=0.069). We believe this could be due to the limited sample size.

Although the HGF/MET pathway seems to be relevant itself in the biology of a subset of SCLCs, cross-talk between the Met receptor and other receptors, such as EGFR or VEGFR has been described [38-40]. Moreover, TK receptor ligands are associated with cancer biology, EMT and progression. As preliminary studies, we explored in these samples circulating levels of some of these ligands at different time points (response assessment and progression). In a subset of these patients, we were able to detect EGF, angiopoietin and VEGF B, C in the serum and more importantly these changed at response and progression (data not shown). These studies are now being completed in the whole series and will be included in another publication.

In summary, we believe that results presented here along with other preclinical and clinical data support the evaluation of Met inhibitors in a selected subpopulation of SCLC patients identified by high sHGF.

## MATERIAL AND METHODS

### Patients and serum samples

This study was an observational study with no intervention. Patients diagnosed with SCLC in our institution were prospectively included. The inclusion criteria were to have a cytohistological diagnosis of SCLC and to sign the informed consent to participate in the study. The first patient was included in January 2010 and the last patient in July 2013. The sample size was calculated based on the assumption that patients with low sHGF would have a 60% 1-year survival and those with high sHGF a 30% (including 10% losses to follow up). Taken these assumptions, 94 patients were necessary to detect differences with 80% statistical power and 0.05 alpha error.

This project was approved by the Local Ethics committee in our institution.

As a control population, we obtained serum samples from age- and sex-matched healthy donors (N: 30) to the study population.

Serum samples from SCLC patients were obtained at diagnosis before starting treatment. All patients that were amenable for treatment received standard first line chemotherapy with a combination of platinum (carboplatin or cisplatin) and etoposide at standard doses. Those patients with stage III or less disease received concomitant radiotherapy with radical intent. All patients with responsive disease subsequently received prophylactic cranial irradiation.

Subsequently, blood samples were obtained at response evaluation (after 3 cycles of chemotherapy for stage IV patients and after chemoradiation for stage III or less). Moreover, at first clinically detected progression, blood samples were collected from patients when available. Patients with at least two samples of serum in two different time points were included in the study.

All clinical and pathologic data was prospectively included in a specific database. Follow up data was also included with a final cut-off point at November 2013.

Previous observations [20, 21] have showed that serum HGF level were significantly higher than the plasma levels. Therefore, for a subset of cases we also obtained plasma samples for comparison.

Serum and plasma blood samples were collected using serum separator tubes (SST) and anticoagulant (EDTA)-coated tubes, respectively. Samples were allowed to clot for 30 minutes before centrifugation for 10 minutes at 1000 g at 4°C. Serum or plasma was removed and assayed immediately or aliquoted and stored at −20°C, according to the protocol (SHG00 Quantikine Human HGF Immunoassay (R&D Systems, Minneapolis, MN).

### HGF ELISA

The Quantikine Human HGF Immunoassay (R&D Systems, Minneapolis, MN) was used to measure HGF levels in human serum [22].

This assay employs the quantitative sandwich immunoassay technique. A monoclonal antibody specific for HGF has been pre-coated onto a microplate. Standards and samples were diluted with the assay diluent, pipetted into the wells and incubated for 2 hours at room temperature. Any HGF present is bound by the immobilized antibody. After washing away any unbound substances, an enzyme-linked polyclonal antibody specific for HGF is added to the wells and incubated for 2 hours at room temperature. Following a wash to remove any unbound antibody-enzyme reagent, a substrate solution is added to the wells and color develops in proportion to the amount of HGF bound in the initial step. The color development is stopped with 2N Sulphuric Acid and the intensity of the color is measured. The optical density of each sample was determined using a microplate reader set at 450 nm. Wavelength correction was set to 540 nm. HGF concentrations were extrapolated from the standard curve generated using the recombinant human HGF of the assay. All samples were run in duplicates.

### Tumor samples and immunohistochemistry

From a subset of patients we were able to analyze by immunohistochemistry in the primary tumor several markers (EMT and p-Met) to study their association with HGF serum levels. Tumor specimens were retrospectively retrieved from Parc de Salut Mar Biobank (MARBiobanc, Barcelona, Spain). Three micrometers tissue sections from formalin-fixed and paraffin embedded samples were obtained, mounted onto charged slides and then, deparaffinised in xylene and hydrated.

The following antibodies were used: MET (SP44) mouse mAb (Ventana-Roche, Tucson, AZ, USA), p-MET Y1234/35 (D26) XP rabbit mAb (Cell Signaling, Danvers, MA, USA), E-cadherin (NCH-38) mouse mAb (Dako, Carpinteria, CA, USA), Snail1 (EC3) mouse mAb, and vimentin (V9) mouse mAb (Dako).

Immunohistochemistry and in situ hybridization for SPARC protocols have been described elsewhere [23]. Stainings were evaluated by two pathologists independently blinded to clinical information on a light microscope (Olympus DX50, Olympus Corp., Tokyo, Japan).

MET, p-MET, and E-cadherin were scored when any percentage of tumor cells was stained in the membrane. Snail1 was evaluated in the nucleus of tumor cells. Vimentin and SPARC were quantified when detected in the cytoplasm of tumor cells. A semiquantitative histoscore (Hscore) was calculated, determined by estimation of the percentage of tumor cells positively stained with low, medium, or high staining intensity for each marker. The final score was determined after applying a weighting factor to each estimate. The formula used was Hscore = (low %) + 2x (medium %) + 3x (high %), and the results ranged from 0 to 300. The tumors in the present study were classified as p-MET, Snail1, SPARC and vimentin negative when the H-score was 0, vs. positive for any positive H-score. For E-Cadherin and total Met, the median was used as the cut-off for positivity.

### Statistical analysis

Statistical analysis was carried out with the R 3.1 program together with the Statistical Assessment Service from IMIM. To analyze associations between categorical variables we used the Chi-square test or the Fisher's exact test as appropriate. Continuous variables were compared with Mann–Whitney U-test. Spearman correlation coefficient was used to assess correlations between HGF from plasma versus serum. Wilcoxon tests were done to compare sHGF levels from patients at different time points. Overall survival was analyzed by Kaplan–Meier method. Curves were compared by the log-rank test. Cox proportional hazards model was used for multivariate analysis. All tests were conducted at the two-sided 0.05 level of significance. This work was performed in accordance with REMARK guidelines [24].

## SUPPLEMENTARY MATERIAL TABLE AND FIGURE


