# Supplementary material for: High circulating hepatocyte growth factor levels associate with epithelial to mesenchymal transition and poor outcome in small cell lung cancer patients
Source: Oncotarget. 2014 Jun 20;5(14):5246–56. doi: 10.18632/oncotarget.2124 (PMC4170595; doi:10.18632/oncotarget.2124)
Supplement: Supplementary file 1 [file oncotarget-05-5246-s001.pdf]

High circulating hepatocyte growth factor levels associate with epithelial to mesenchymal transition and poor outcome in small cell lung cancer patients

Supplementary Material

Supplementary Table 1: Biomarker expression in tumor samples

Biomarker	Met	p-Met	Snail1	Vimentin	SPARC	E-cadherin
Positive (%)	22 (49)	14 (32)	12 (28)	10 (23)	11 (25)	27 (60)
Negative (%)	23 (51)	30 (68)	31 (72)	33 (77)	33 (75)	18 (40)

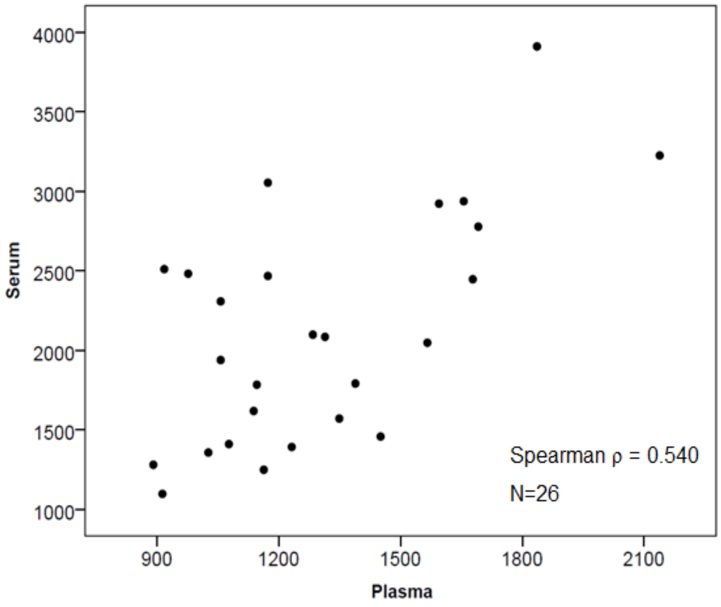

Supp figure 1: Correlation of serum and plasma HGF measurements. Spearman correlation analysis was performed to evaluate differences between HGF plasma and serum values from 26 paired, baseline patient samples. Spearman rho and the number of patients are displayed in the graph.
